# Comparative transcriptome analysis reveals the transcriptional alterations in heat-resistant and heat-sensitive sweet maize (*Zea mays* L.) varieties under heat stress

**DOI:** 10.1186/s12870-017-0973-y

**Published:** 2017-01-25

**Authors:** Jiang Shi, Baiyuan Yan, Xuping Lou, Huasheng Ma, Songlin Ruan

**Affiliations:** 1 Institute of Crop Science, Hangzhou Academy of Agricultural Sciences, Hangzhou, 310024 People’s Republic of China; 2 Laboratory of Plant Molecular Biology & Proteomics, Institute of Biotechnology, Hangzhou Academy of Agricultural Sciences, Hangzhou, 310024 People’s Republic of China; 3 Jiande seed management station, Hangzhou, 311600 People’s Republic of China; 4 Xianshan Institute of Agricultural Sciences, Hangzhou, 330100 People’s Republic of China

**Keywords:** Sweet maize, Heat-resistance, Transcriptome profiling, Gene Ontology, Pathway analysis

## Abstract

**Background:**

Despite the heat-related physiology and heat-shock proteins in maize have been extensively studied, little is known about the transcriptome profiling of how the maize varieties with different genotypes responding to high temperatures. Seedling mortality of Xiantian 5 (XT) is significantly lower than that of Zhefengtian (ZF) when exposed to high temperature (42 °C for 6 h) and followed by a recovery growth (25 °C for one week). Therefore, we performed a transcriptome analysis using the total RNA extracted from the leaves of XT and ZF that were previously subjected to heat stress at 42 °C for 0 h, 0.5 h, and 3 h, respectively.

**Results:**

A total of 516 commonly up-regulated and 1,261 commonly down-regulated genes were identified among XT/ZF, XT0.5/ZF0.5 and XT3/ZF3 using transcriptome analysis. Gene Ontology classification of the 516 up-regulated genes showed that their encoded proteins were significantly assigned to 18 cellular components, and were classified into 9 functional categories, and were involved in 9 biological processes. Most of proteins encoded by up-regulated genes were localized in chloroplast and its structural components, and involved in multiple biological processes associated with photosynthesis, indicating that these chloroplast proteins play an important role in increasing heat tolerance in sweet maize. While the proteins encoded by 1,261 down-regulated genes were significantly assigned to 31 cellular components, and were classified into 3 functional categories, and were involved in 9 biological processes. Interestingly, these proteins were involved in a series of biological processes from gene expression to translation, suggesting that lowering these processes may contribute to improved heat resistance in sweet maize. The up-regulated genes were identified to be involved in 36 distinct metabolic pathways, of which the most significant ones was secondary metabolite biosynthetic pathway. While the down-regulated genes were identified to be involved in 23 distinct metabolic pathways, of which the most significant ones were found in ribosome. Quantitative real-time PCR analysis demonstrated that 5 genes involved in the biosynthesis of secondary metabolites and photosynthesis in XT have higher abundance than those in ZF, whereas 5 ribosome genes in XT showed lower abundance than those in ZF. In addition, heat-tolerant sweet maize may keep at lower growth level than heat-sensitive one through dowregulating expression of genes related to zeatin and brassinosteroid biosynthesis to better regulate heat stress responses.

**Conclusions:**

Comparative transcriptomic profiling reveals transcriptional alterations in heat-resistant and heat-sensitive sweet maize varieties under heat stress, which provides a new insight into underlying molecular mechanism of maize in response to heat stress.

**Electronic supplementary material:**

The online version of this article (doi:10.1186/s12870-017-0973-y) contains supplementary material, which is available to authorized users.

## Background

Maize originates from the highlands of Central and South America’s tropical and subtropical regions and is adapted to warm temperatures [1]. Although climatic factors, such as light, temperature, water, and CO_2_ in air, all have significant influences on maize production, temperature is still the major factor affecting maize growth and development [1]. In recent years, with the increasing and frequent occurrence of extremely high temperatures due to global warming, high temperature has become one of the most important abiotic stresses restricting crop production worldwide [2]. Heat stress affects maize flowering, pollination, and grain filling, which then results in the decline of seed setting rate and thus reduces maize production [1]. Therefore, the adverse effect of high temperatures on maize production is increasingly becoming a concern.

Maize seedlings grown under high temperatures for long durations will have thin leaf morphology, and their leaf colors gradually change from green to light green, and eventually become yellow. Heat stress can cause the reductions in leaf extension rate, shoot biomass, and CO_2_ assimilation rate [3]. High temperature during the flowering stage can lead to reduced pollen quality, low yield, and poor quality of the final products [1]. Further research shows that heat stress can affect grain crude protein, crude fat, and lysine contents, which in turn leads to the low quality of maize products [4]. In addition, heat-resistant maize variety maintains higher levels of chlorophyll content, photosystem II electron transfer rate, photosynthetic rate, and other important physical characteristics under heat stress [4].

The molecular mechanisms underlying plant heat tolerance including the alteration of signaling cascades and transcriptional control, increasing production of antioxidants [5, 6] and osmoprotectants, and the expression of heat shock proteins [7], have been presented. Heat shock proteins (HSPs) are a type of proteins with highly conserved amino acid sequences and functions. HSPs function as molecular chaperones and are involved in repairing and refolding damaged proteins as well as synthesizing, folding and transporting normal proteins [8]. Extensive studies have demonstrated the notable protection of HSP70, HSP101 and smHSPs family proteins from heat stress.

Transcriptomics is a powerful tool for discovering differentially expressed genes and has been widely applied in some crop species, including rice [9–12], maize [13], wheat [14], barley [15], cotton [16, 17], rape [18], potato [19], tea [20], tomato [21], pepper [22], watermelon [23], *Phaseolus vulgaris* [24], *Vigna mungo* [24], pea [25], chickpea [26] and citrus fruit [27]. Among them, the transcriptome profiling of rice [9, 10], barley [15], pepper [22] and maize [28] in response to heat stress has been performed. However, comparative transcriptome analysis has only been performed in rice and pepper between heat-resistant and heat-sensitive cultivars. In this study, to detect the differential gene expression in different maize genotypes under heat stress, heat-resistant and heat-sensitive maize seedlings were treated at 42 °C, and the expression of genes in leaves collected at different time points was measured. A comparative transcriptomic analysis was performed to reveal the significantly up-regulated and down-regulated genes. Gene Ontology (GO) classification of the proteins encoded by these genes was used to analyze their cellular locations. A pathway analysis was performed to reveal the biological pathways involving these genes. This study may provide a new insight into the transcriptional alterations in heat-resistant and heat-sensitive sweet maize varieties responding to heat stress.

## Results

### Responses of maize seedlings with different genotypes to heat stress

**Fig. 1:**
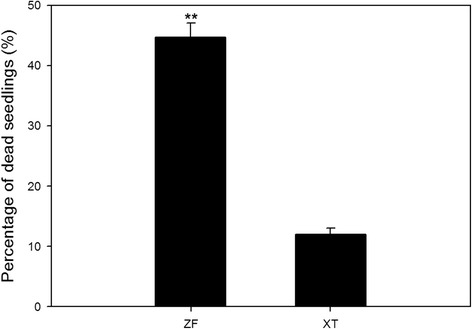
Mortality of maize seedlings of different genotypes under heat stress. Maize varieties XT (heat-resistant) and ZF (heat-sensitive) were treated at 42 °C for 6 h, followed by recovery growth at 25 °C for one week. Three independent experimental replicates were analyzed for each sample, and data were indicated as mean ± SE (*n* = 3). XT: Xiantian 5; ZF: Zhefengtian 2

Maize varieties XT (heat-resistant) and ZF (heat-sensitive) were treated with the high temperature of 42 °C, followed by one week of recovery growth at 25 °C. As shown in Fig. 1, the seedling mortality of XT was significantly lower than that of ZF, indicating that XT is more resistant to heat stress.

### Gene expression profiles of different maize genotypes in response to heat stress

**Fig. 2:**
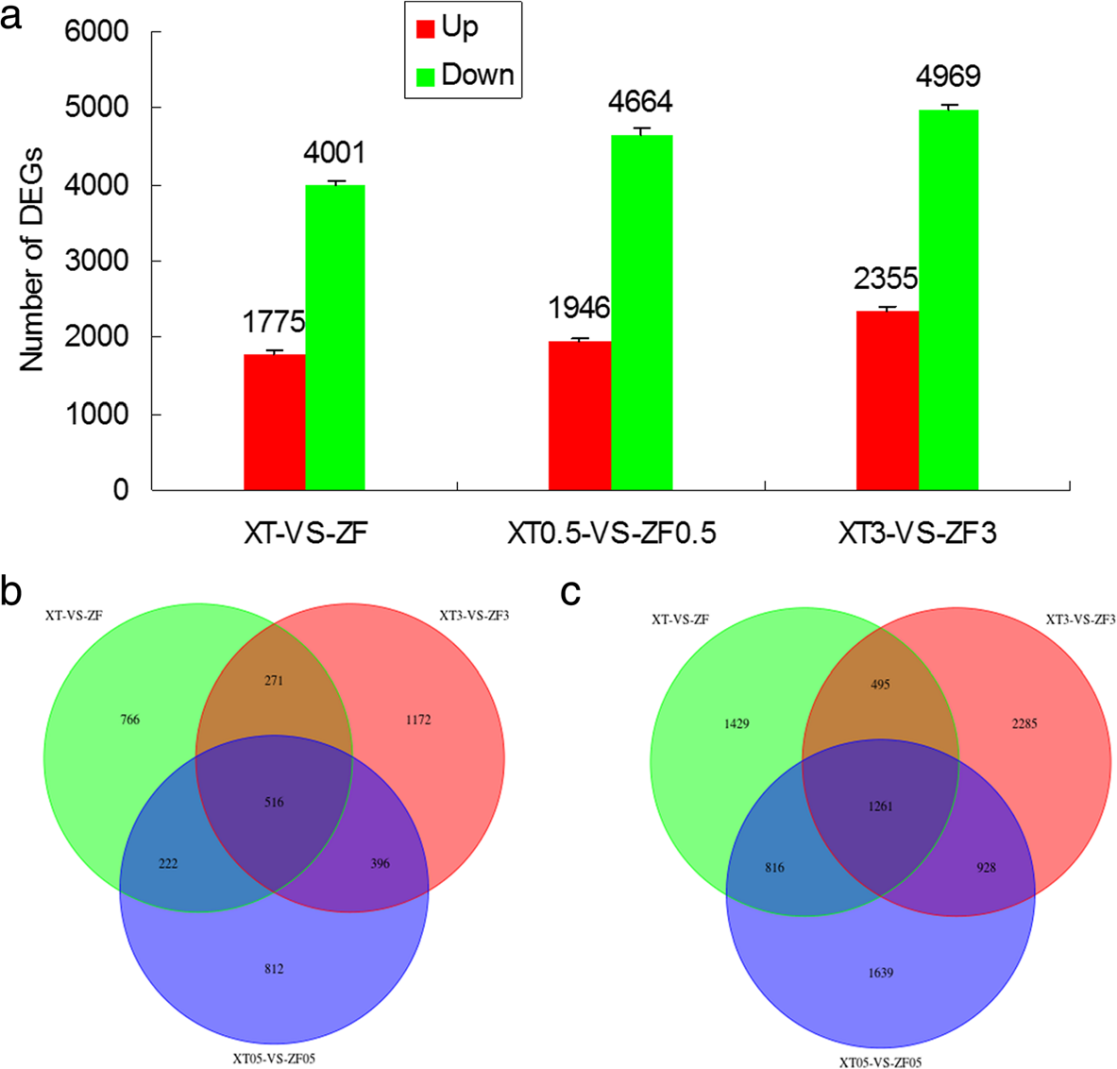
Gene expression profile of different maize genotypes in response to heat stress. **a** The total number of up-regulated and down-regulated genes. **b** Venn diagram of up-regulated genes. **c** Venn diagram of down-regulated genes. Three independent experimental replicates were analyzed for each sample, and data were indicated as mean ± SE (*n* = 3). XT: Xiantian 5; ZF: Zhefengtian 2. XT-ZF, XT0.5 –ZF0.5 and XT3-ZF3 represent XT-ZF seedlings treated at 42 °C for 0, 0.5, 3 h, respectively

As shown in Fig. 2a, different from the heat sensitive variety ZF, the maize variety XT (heat-resistant) showed an increased number of differentially expressed genes under different durations of heat treatment (0, 0.5 h or 3 h), including both up-regulated and down-regulated genes. The differential expression analysis of XT/ZF, XT0.5/ZF0.5 and XT3/ZF3 identified 516 commonly up-regulated and 1,261 commonly down-regulated genes (Fig.2b and c). In addition, the number of uniquely up-regulated or down-regulated genes between XT/ZF, XT0.5/ZF0.5 or XT3/ZF3 was increased with increasing duration of heat treatment. There were 766, 812, and 1,172 down-regulated genes, and 1,429, 1,639, and 2,285 up-regulated genes, respectively (Fig.2b and c).

### GO classification of common differential genes

We then performed a GO classification of 516 up-regulated genes, and the results showed that the proteins encoded by these genes were significantly assigned to 18 cellular components including thylakoid part (GO: 0044436), photosynthetic membrane (GO: 0034357), chloroplast thylakoid (GO: 0009534), plastid thylakoid (GO: 0031976), thylakoid membrane (GO: 0042651), thylakoid (GO: 0009579), organelle subcompartment (GO: 0031984), plastid thylakoid membrane (GO: 0055035), plastid part (GO: 0044435), thylakoid lumen (GO: 0031977), chloroplast thylakoid membrane (GO: 0009535), photosystem (GO: 0009521), chloroplast part (GO: 0044434), chloroplast (GO: 0009507), photosystem II (GO: 0009523), plastid (GO: 0009536), photosystem I (GO: 0009522) and envelope (GO: 0031975) (Table 1). Subsequently, proteins encoded by the up-regulated genes were classified into 9 functional categories, including 50 proteins with oxidoreductase activity (GO: 0016491), 6 proteins with peptidase inhibitor activity (GO: 0030414), 6 proteins with peptidase regulator activity (GO: 0061134), 18 proteins with tetrapyrrole binding (GO: 0046906), 4 proteins with inositol-1,3,4-trisphosphate 6-kinase activity (GO: 0052725), 4 proteins with inositol tetrakisphosphate kinase activity (GO: 0051765), 4 proteins with inositol trisphosphate kinase activity (GO: 0051766), 16 proteins with heme binding (GO: 0020037) and 2 proteins with omega-3 fatty acid desaturase activity (GO: 0042389) (Table 1). Finally, they were assigned to be mainly involved in 9 biological processes, photosynthesis (GO: 0015979), oxidation-reduction process (GO: 0055114), photosynthesis, light reaction (GO: 0019684), negative regulation of peptidase activity (GO: 0010466), regulation of peptidase activity (GO: 0052547), negative regulation of hydrolase activity (GO: 0051346), regulation of proteolysis (GO: 0030162), regulation of protein processing (GO: 0070613) and regulation of protein metabolic process (GO: 0051246) (Table 1).

**Table 1:** GO classification of common up-regulated genes in both XT and ZF

Gene Ontology term	The number of Genes	-log_10_ (*P* value)*
Cellular component		
thylakoid part (GO: 0044436)	26	9.16494
photosynthetic membrane (GO: 0034357)	22	6.8697
chloroplast thylakoid (GO: 0009534)	24	6.6383
plastid thylakoid (GO: 0031976)	24	6.6253
thylakoid membrane (GO: 0042651)	21	6.3925
Thylakoid (GO: 0009579)	27	6.1649
organelle subcompartment (GO: 0031984)	24	6.1524
plastid thylakoid membrane (GO: 0055035)	19	5.4935
plastid part (GO: 0044435)	47	5.3468
thylakoid lumen (GO: 0031977)	10	5.1169
chloroplast thylakoid membrane (GO: 0009535)	18	4.8125
photosystem (GO: 0009521)	9	4.8125
chloroplast part (GO: 0044434)	45	4.7011
chloroplast (GO: 0009507)	67	4.1675
photosystem II (GO: 0009523)	6	2.7447
plastid (GO: 0009536)	98	2.6253
photosystem I (GO: 0009522)	5	2.0477
envelope (GO: 0031975)	4	1.3821
Molecular function		
oxidoreductase activity (GO: 0016491)	50	2.9031
peptidase inhibitor activity (GO: 0030414)	6	2.7447
peptidase regulator activity (GO: 0061134)	6	2.7447
tetrapyrrole binding (GO: 0046906)	18	2.5031
inositol-1,3,4-trisphosphate 6-kinase activity (GO: 0052725)	4	1.9119
inositol tetrakisphosphate kinase activity (GO: 0051765)	4	1.7755
inositol trisphosphate kinase activity (GO: 0051766)	4	1.7113
heme binding (GO: 0020037)	16	1.600
omega-3 fatty acid desaturase activity (GO: 0042389)	2	1.3372
Biological process		
photosynthesis (GO: 0015979)	16	4.0996
oxidation-reduction process (GO: 0055114)	57	3.7959
photosynthesis, light reaction (GO: 0019684)	10	3.0132
negative regulation of peptidase activity (GO: 0010466)	6	2.3615
regulation of peptidase activity (GO: 0052547)	6	2.3615
negative regulation of hydrolase activity (GO: 0051346)	6	2.2660
regulation of proteolysis (GO: 0030162)	6	1.9618
regulation of protein processing (GO: 0070613)	6	1.96182
regulation of protein metabolic process (GO: 0051246)	15	1.3416

*P values of all GO terms are lower than 0.05. Conversely, −log_10_ (*P* value) values of all GO terms are greater than 1.3010, that is, the greater -log_10_ (*P* value) value, the better significance

Similarly, we performed a GO classification of 1,261 commonly down-regulated genes, and discovered that the proteins encoded by these genes were significantly assigned to 31 cellular components, including ribosomal subunit (GO: 0044391), cytosolic ribosome (GO: 0022626), cytosolic part (GO: 0044445), ribosome (GO: 0005840), ribonucleoprotein complex (GO: 0030529), cytosolic large ribosomal subunit (GO: 0022625), large ribosomal subunit (GO: 0015934), nucleolus (GO: 0005730), cytosol (GO: 0005829), membrane-enclosed lumen (GO: 0031974), cytosolic small ribosomal subunit (GO: 0022627), organelle lumen (GO: 0043233), intracellular organelle lumen (GO: 0070013), small ribosomal subunit (GO: 0015935), nuclear lumen (GO: 0031981), non-membrane-bounded organelle (GO: 0043228), intracellular non-membrane-bounded organelle (GO: 0043232), nuclear part (GO: 0044428), intracellular organelle part (GO: 0044446), organelle part (GO: 0044422), vacuolar membrane (GO: 0005774), vacuolar part (GO: 0044437), cytoplasm (GO: 0005737), chloroplast (GO: 0009507), cell-cell junction (GO: 0005911), plasmodesma (GO: 0009506), cell junction (GO: 0030054), symplast (GO: 0055044), vacuole (GO: 0005773), macromolecular complex (GO: 0032991) and cytoplasmic part (GO: 0044444) (Table 2). Next, proteins encoded by the down-regulated genes were classified into 3 functional categories, including 66 proteins with structural constituent of ribosome (GO: 0003735), 70 proteins structural molecule activity (GO: 0005198) and 3 proteins with glutamate-cysteine ligase activity (GO: 0004357) (Table 2). Finally, they were assigned to be involved in 14 biological processes, including translation (GO: 0006412), gene expression (GO: 0010467), cellular macromolecule biosynthetic process (GO: 0034645), macromolecule biosynthetic process (GO: 0009059), cellular biosynthetic process (GO: 0044249), biosynthetic process (GO: 0009058), organic substance biosynthetic process (GO: 1901576), ribosome biogenesis (GO: 0042254), metabolic process (GO: 0042254), ribonucleoprotein complex biogenesis (GO: 0022613), polysaccharide localization (GO: 0033037), callose localization (GO: 0052545), sulfur compound metabolic process (GO: 0006790) and defense response by callose deposition (GO: 0052542) (Table 2).

**Table 2:** GO classification of common down-regulated genes in both XT and ZF

Gene Ontology term	The number of Genes	-log_10_ (*P* value)*
Cellular component		
ribosomal subunit (GO: 0044391)	59	25.0200
cytosolic ribosome (GO: 0022626)	67	23.1024
cytosolic part (GO: 0044445)	69	21.3206
ribosome (GO: 0005840)	77	20.5086
ribonucleoprotein complex (GO: 0030529)	91	19.3747
cytosolic large ribosomal subunit (GO: 0022625)	34	14.6536
large ribosomal subunit (GO: 0015934)	34	13.6498
nucleolus (GO: 0005730)	65	12.4437
cytosol (GO: 0005829)	157	10.3215
membrane-enclosed lumen (GO: 0031974)	88	10.2774
cytosolic small ribosomal subunit (GO: 0022627)	24	9.7670
organelle lumen (GO: 0043233)	86	9.6556
intracellular organelle lumen (GO: 0070013)	86	9.6556
small ribosomal subunit (GO: 0015935)	25	9.4776
nuclear lumen (GO: 0031981)	80	9.4168
non-membrane-bounded organelle (GO: 0043228)	118	8.3089
intracellular non-membrane-bounded organelle (GO: 0043232)	118	8.3089
nuclear part (GO: 0044428)	84	6.6144
intracellular organelle part (GO: 0044446)	233	5.6180
organelle part (GO: 0044422)	233	5.5031
vacuolar membrane (GO: 0005774)	60	4.1891
vacuolar part (GO: 0044437)	60	4.0645
cytoplasm (GO: 0005737)	553	4.0376
chloroplast (GO: 0009507)	140	4.0000
cell-cell junction (GO: 0005911)	75	2.2676
plasmodesma (GO: 0009506)	75	2.2676
cell junction (GO: 0030054)	75	2.2676
symplast (GO: 0055044)	75	2.2676
vacuole (GO: 0005773)	75	1.8658
macromolecular complex (GO: 0032991)	135	1.7261
cytoplasmic part (GO: 0044444)	519	1.6070
Molecular function		
structural constituent of ribosome (GO: 0003735)	66	22.5045
structural molecule activity (GO: 0005198)	70	16.4921
glutamate-cysteine ligase activity (GO: 0004357)	3	1.8413
Biological process		
translation (GO: 0006412)	80	14.2749
gene expression (GO: 0010467)	125	8.8182
cellular macromolecule biosynthetic process (GO: 0034645)	112	5.7328
macromolecule biosynthetic process (GO: 0009059)	112	5.3316
cellular biosynthetic process (GO: 0044249)	175	5.1952
biosynthetic process (GO: 0009058)	187	4.7235
organic substance biosynthetic process (GO: 1901576)	175	3.8861
ribosome biogenesis (GO: 0042254)	24	3.8239
metabolic process (GO: 0042254)	580	2.9788
ribonucleoprotein complex biogenesis (GO: 0022613)	26	2.5768
polysaccharide localization (GO: 0033037)	9	2.2790
callose localization (GO: 0052545)	9	2.2790
sulfur compound metabolic process (GO: 0006790)	19	1.4773
defense response by callose deposition (GO: 0052542)	7	1.4176

*P values of all GO terms are lower than 0.05. Conversely, −log_10_ (*P* value) values of all GO terms are greater than 1.3010, that is, the greater -log_10_ (*P* value) value, the better significance

### Pathway analysis of common differential genes

**Fig. 3:**
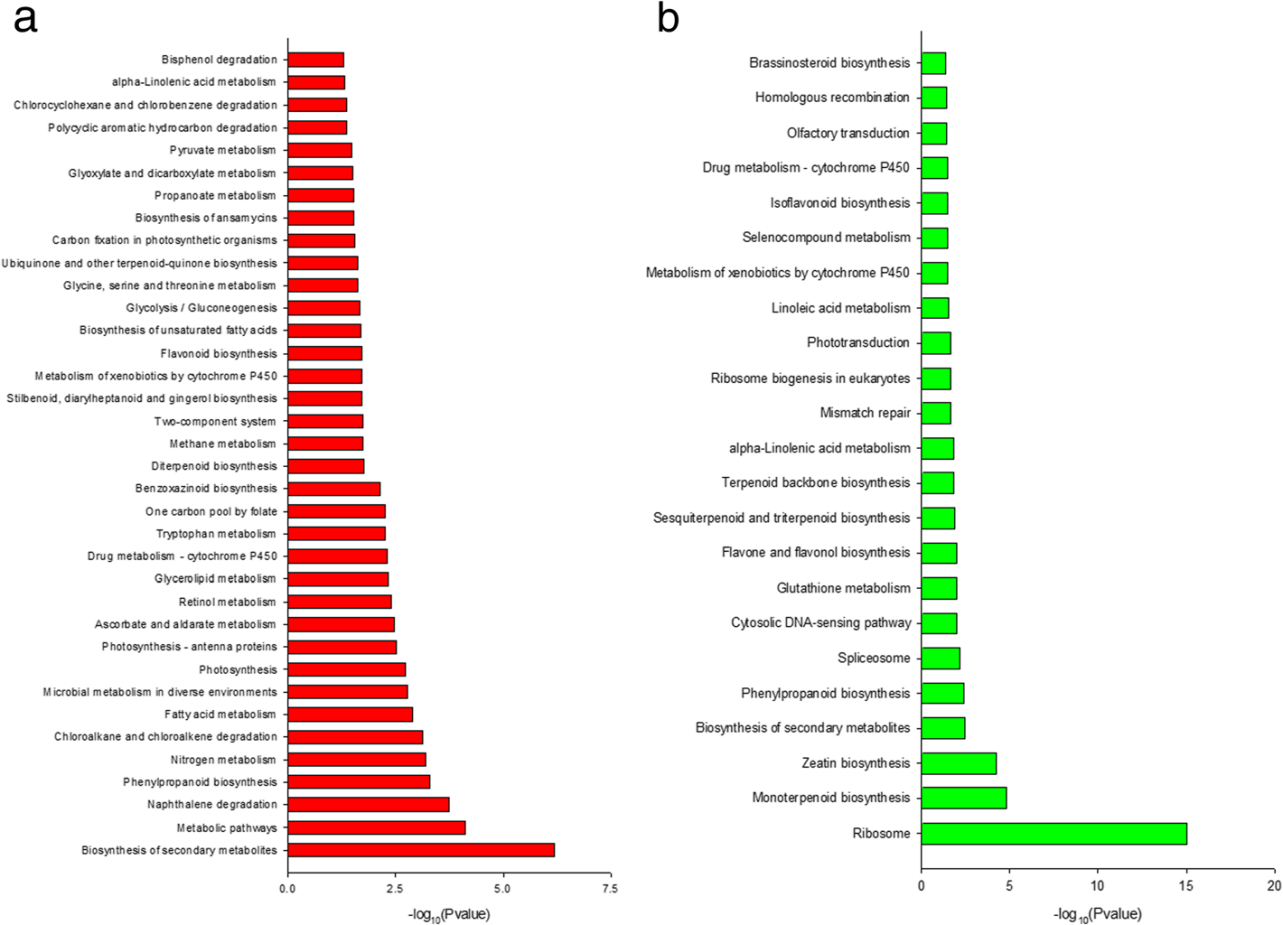
KEGG pathway enrichment analysis based on the differentially expressed genes. **a** Pathway enrichment analysis based on the differentially up-regulated genes in both XT and ZF. **b** Pathway enrichment analysis based on the differentially down-regulated genes in both XT and ZF. XT: Xiantian 5; ZF: Zhefengtian 2

To determine the involvement of these differentially expressed genes in heat resistance, we performed a pathway analysis to identify the potential target genes (Fig. 3). The up-regulated genes have been identified to be involved in 36 distinct metabolic pathways, including biosynthesis of secondary metabolites, metabolic pathway, fatty acid metabolism, microbial metabolism in diverse environments, photosynthesis, photosynthesis - antenna proteins, ascorbate and aldarate metabolism, retinol metabolism, glycerolipid metabolism, drug metabolism - cytochrome P450, tryptophan metabolism, one carbon pool by folate, benzoxazinoid biosynthesis, diterpenoid biosynthesis, methane metabolism, two-component system, stilbenoid, diarylheptanoid and gingerol biosynthesis, metabolism of xenobiotics by cytochrome P450, flavonoid biosynthesis, biosynthesis of unsaturated fatty acids, glycolysis/gluconeogenesis, glycine, serine and threonine metabolism, ubiquinone and other terpenoid-quinone biosynthesis, carbon fixation in photosynthetic organisms, biosynthesis of ansamycins, propanoate metabolism, glyoxylate and dicarboxylate metabolism, pyruvate metabolism, polycyclic aromatic hydrocarbon degradation, chlorocyclohexane and chlorobenzene degradation, alpha-Linolenic acid metabolism, and bisphenol degradation (Fig. 3a). Among them, the most significant ones were secondary metabolite biosynthetic pathway, followed by the metabolic pathway. In addition, some other pathways were involved in photosynthesis. While the downregulated genes have been identified to be involved in 23 distinct metabolic pathways, including ribosome, zeatin biosynthesis, biosynthesis of secondary metabolites, phenylpropanoid biosynthesis, spliceosome, cytosolic DNA-sensing pathway, glutathione metabolism, sesquiterpenoid and triterpenoid biosynthesis, terpenoid backbone biosynthesis, alpha-Linolenic acid metabolism, mismatch repair, ribosome biogenesis in eukaryotes, phototransduction, linoleic acid metabolism, metabolism of xenobiotics by cytochrome P450, selenocompound metabolism, isoflavonoid biosynthesis, drug metabolism-cytochrome P450, olfactory transduction, homologous recombination, and brassinosteroid biosynthesis (Fig. 3b). Among them, the most significant ones were found in ribosome, and the other pathways were related to monoterpenoid biosynthesis and zeatin biosynthesis.

### Validation of differentially expressed candidate genes

**Fig. 4:**
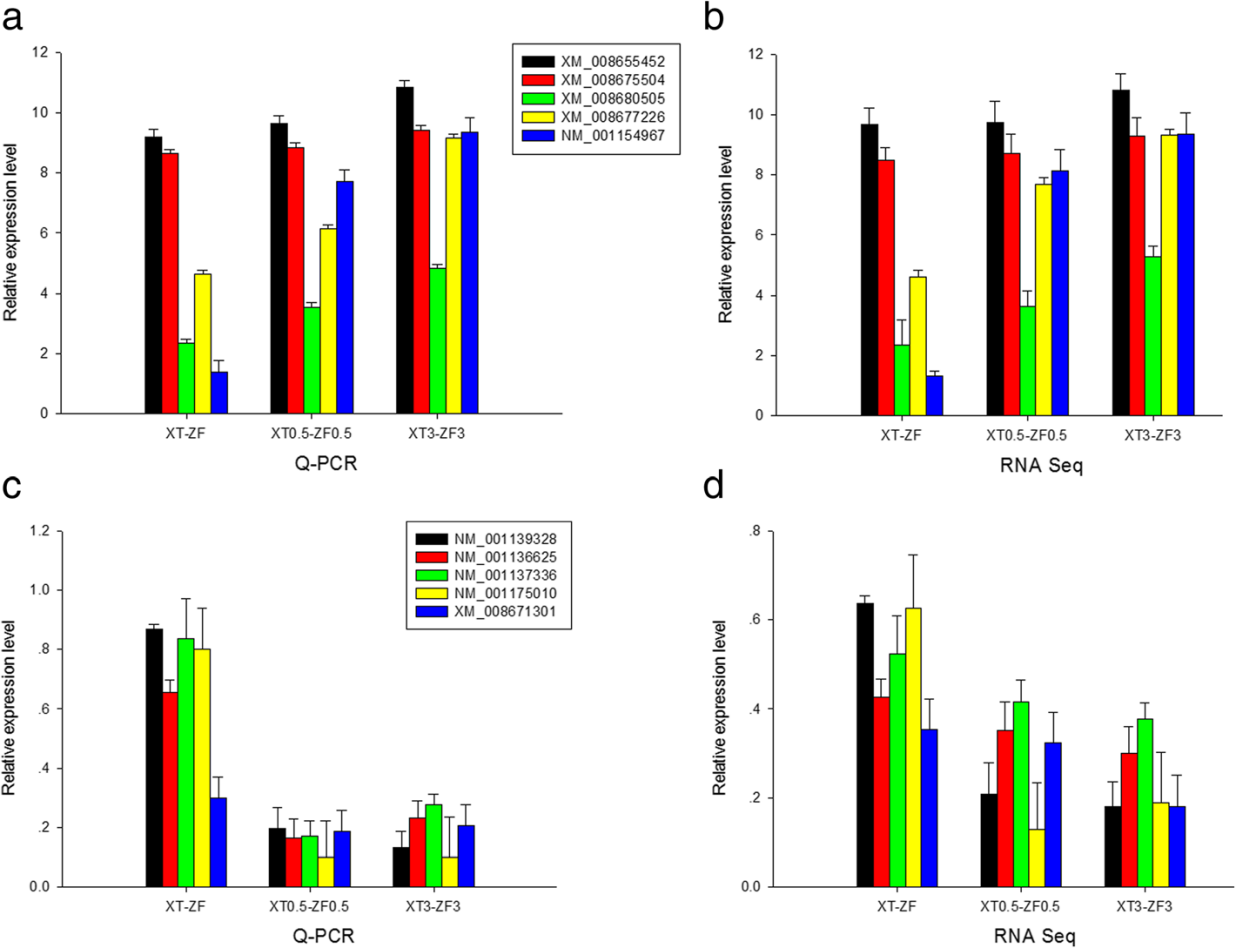
Validation of differentially expressed candidate genes. **a** qRT-PCR analysis of five up-regulated genes in response to heat stress in XT and ZF. **b** Expression of five up-regulated genes in XT and ZF based on RNA-seq data. **c** qRT-PCR analysis of five down-regulated genes in response to heat stress in XT and ZF. **d** Expression of five down-regulated genes in XT and ZF based on RNA-seq data. Three independent experimental replicates were analyzed for each sample, and data were indicated as mean ± SE (*n* = 3)

To validate the Illumina sequencing data and the expression patterns of the DEGs revealed by RNA-Seq, qRT-PCR was performed to examine the expression patterns of 10 DEGs, including 5 genes involved in the biosynthesis of secondary metabolites and photosynthesis, and 5 ribosome genes (Fig. 4). qRT-PCR results showed that 5 genes involved in the biosynthesis of secondary metabolites and photosynthesis, including XM_008655452 (pyruvate decarboxylase 3-like), XM_008675504 (uncharacterized LOC103649793), XM_008680505 (psbQ-like protein 1, chloroplastic), XM_008677226 (chlorophyll a-b binding protein of LHCII type 1-like) and NM_001154967 (chlorophyll a-b binding protein 2), in XT had higher abundance than those in ZF (Fig. 4a), while 5 ribosome genes, including NM_001139328 (60S ribosomal protein L32), NM_001136625 (60S ribosomal protein L7a), NM_001137336 (ribosomal protein L13A-like protein), NM_001175010 (Ribosomal protein L3) and XM_008671301 (60S ribosomal protein L37a) in XT showed lower abundance than those in ZF (Fig. 4c), which was consistent with the RNA-seq data from XT and ZF (Fig. 4b and d).

## Discussion

High temperature is an adverse factor influencing both plant growth and development, thereby causing extensive loss of yield [29]. Although the physiological effects of heat stress on crops has been extensively reported, the understanding of underlying molecular mechanism remains limited. In the present study, we found that most of proteins encoded by up-regulated genes were localized in chloroplast and its structural components, and involved in multiple biological processes associated with photosynthesis. Obviously, they are closely related to function of chloroplast, especially photosynthesis, indicating that these chloroplast proteins play an important role in increasing heat tolerance in sweet maize. In contrast, the proteins encoded by 1,261 down-regulated genes were localized in multiple cellular components, including cytoplasm, nuclear, non-membrane-bounded organelle, ribosome, vacuole, chloroplast and plasmodesma, and were involved in a series of biological processes from gene expression to translation, suggesting that lowering these processes may contribute to improved heat resistance in sweet maize.

Here, we found there was an apparent connection between the heat tolerance of sweet corn and the alterations in 3 pathways, including the biosynthesis of secondary metabolites, photosynthesis (up-regulation) and ribosome function (down-regulation), which was consistent with the results of previous studies [14, 21]. Apart from the above-mentioned pathways, the differences in 9 pathways including photosynthesis, photosynthesis-antenna proteins, stilbenoid, diarylheptanoid and gingerol biosynthesis, flavonoid biosynthesis, diterpenoid biosynthesis, biosynthesis of unsaturated fatty acids, nitrogen metabolism, flavone and flavonol biosynthesis and monoterpenoid biosynthesis, were found between heat-tolerance and heat-sensitive sweet maize cultivars, which also appeared between heat-tolerance and heat-sensitive pepper ones [22], indicating that they might be the most fundamental pathways involved in heat tolerance in other crop species.

Interestingly, both up-regulated and down-regulated genes have been identified to be involved in 5 identical pathways including biosynthesis of secondary metabolites, phenylpropanoid biosynthesis, alpha-Linolenic acid metabolism, metabolism of xenobiotics by cytochrome P450 and drug metabolism- cytochrome P450, indicating that genes involved in these pathways showed patterns of both upregulating and downregulating expression, which was likely to help keep these pathways in balance under heat stress.

Previous studies showed that 7 hormones including ABA, auxin, jasmonic acid (JA), cytokinins (CKs), ethylene, gibberellin, and brassinosteroid were likely to be involved in heat stress [22]. Interestingly, several hormones including ABA, brassinosteroids (BRs), and ethylene possibly interacted through complex networks to regulate heat stress responses [30]. In the present study, we found that some genes related to two plant hormone signal pathways including zeatin biosynthesis and brassinosteroid biosynthesis had lower levels in XT (heat tolerant) than in ZF (heat sensitive), indicating that reduced biosynthesis of zeatin and brassinosteroid was likely to be related to heat tolerance in sweet maize. Accumulating evidences also demonstrated that changes in zeatin content were related to plant heat tolerance. In creeping bentgrass, the levels of various cytokinins, zeatin (Z), zeatin riboside (ZR), dihydrogen zeatin riboside (DHZR) and isopentinyl adenosine (iPA), showed dramatic decline in root and shoot under high soil temperature, which were correlated with decreased dry matter production [31]. In a dwarf wheat variety, high-temperature-induced decrease in cytokinin content was responsible for reduced kernel filling and its dry weight [32]. However, brassinosteroids conferred the basic thermotolerance to tomato and oilseed rape (*Brassica napus*), but not to cereals [33]. Therefore, it was suggested that heat-tolerant sweet maize might keep at lower growth level than heat-sensitive one through dowregulating expression of genes related to zeatin and brassinosteroid biosynthesis to better regulate heat stress responses.

## Conclusions

Comparative transcriptome analsis revealed 516 commonly up-regulated and 1,261 commonly down-regulated genes between heat tolerant and heat sensitive sweet maize genotypes under heat stress. Gene Ontology classification and KEGG pathway analysis of these differentially expressed genes showed that secondary metabolite biosynthetic pathway and ribosome were the most significant ones. Further analysis revealed that 9 fundamental pathways, 5 identical pathways and 2 hormonal signal pathways (zeatin and brassinosteroid biosynthesis) were likely to play important roles in regulating the response of maize to heat stress. Therefore, our results provide a new insight into transcriptional alterations in heat-resistant and heat-sensitive sweet maize varieties under heat stress, which helps to address underlying molecular mechanism of maize in response to heat stress.

## Methods

### Plant materials, growth conditions and heat treatment

Sweet maize seeds of Xiantian 5 (XT) and Zhefengtian 2 (ZF) were supplied by the Zhejiang Wuwangnong Seed Group Co., Ltd. (Hangzhou, Zhejiang province, China). Four replicates of 50 seeds for each treatment and each genotype were placed in the germination boxes (18 cm × 13 cm × 10 cm) containing a layer of moistened peat matrix (30 mm in thickness), and then surface covered with a thin layer of peat matrix. The seeds were germinated for 21 days at 25 °C, 90% relative humidity, and 16 h light/8 h dark. The three-week-old seedlings were treated with 42 °C for 0.5 h, 3 h, and 6 h, respectively. Maize leaves experiencing 42 °C heat stress for 0.5 h or 3 h were collected and subsequently used for transcriptomic analysis. Maize seedlings receiving 42 °C treatment for 6 h were cultivated in a 25 °C incubator with 90% relative humidity and 16 h light/8 h dark. Mortality of these seedlings were determined 7 days after incubation at 25 °C, 90% relative humidity, and 16 h light/8 h dark.

### RNA sequencing and data analysis

Maize leaves from ten plants were pooled as an independent experimental replicate, and the leaves from other ten plants that were treated in the same growth chamber at intervals of three weeks were pooled as another independent experimental replicate. Three independent experimental replicates were used for transcriptomic analysis. Total leaf RNA was isolated from maize leaves using Trizol reagent (Invitrogen, USA) according to the manufacturer’s protocols, dissolved in RNase-free water and then used to construct transcriptome sequence library using the NEBNext Ultra RNA Library Prep Kits for Illumina (NEB, USA) following the manufacturer’s instructions. Index codes were added to attribute sequences to each sample. At last, 125 bp paired-end reads were generated using Illumina HiSeq 2500 (Novogene, China). Clean reads were obtained by removing the reads containing adapter or ploy-N and the low quality reads from raw data. They were aligned to the B73 maize genome using the TopHat (2.0.9) software. To measure gene expression level, the total number of reads per kilobases per millionreads (RPKM) of each gene was calculated based on the length of this gene and the counts of reads mapped to this gene. RPKM values were calculated based on all the uniquely mapped reads. The genes with RPKM ranging from 0 to 3 were considered at a low expression level; the genes with RPKM ranging from 3 to 15 at a medium expression level; and the genes with RPKM above 15 at a high expression level. Differential expression analysis was calculated using the DESeq R package (1.10.1). The resulting *p* values were adjusted using the Benjamini and Hochberg’s approach for controlling the false discovery rate. Genes with an adjusted p value <0.05 identified by DESeq were assigned as differentially expressed. GO annotation was performed using the Blast2GO software (GO association was done by a BLASTX against the NCBI NR database). GO enrichment analysis of differentially expressed genes was then performed by the BiNGO plugin for Cytoscape. Over-presented GO terms were identified using a hypergeometric test with the significance threshold of 0.05 after the Benjamini and Hochberg FDR correction. KEGG enrichment analysis of differentially expressed genes was performed using the KOBAS (2.0) [34] software.

### Verification of RNA-seq data by quantitative real-time PCR (qRT-PCR)

To test the reliability of RNA-seq data (Additional file 1), a set of top ten up-regulated genes in three replicates were selected for qRT-PCR. Specific primers were designed with the Primer Express software (Applied Biosystems) and synthesized by Sangon (Shanghai, China). cDNA was synthesized from 1 μg of total RNA using the PrimeScript RT reagent Kit (Takara, Dalian, China). Real-time RT-PCR was performed on the ABI 7500 Real-Time PCR System (Applied Biosystems) using the 2× SYBR green PCR master mix (Applied Biosystems). Three independent experimental replicates were analyzed for each sample, and data were indicated as mean ± SE (*n* = 3). Eleven pairs of primers were designed for gene-specific transcript amplification (Additional file 2).

## Additional files

Additional file 1: RNA-seq dataset of maize seedlings of different genotypes under heat stress. (XLS 18900 kb)
Additional file 2: Eleven pairs of primers were designed for gene-specific transcript amplification. (DOC 28 kb)

## Abbreviations

ABA: Abscisic acid
BRs: Brassinosteroids
CKs: Cytokinins
DEGs: Differential expression genes
GO: Gene Ontology
HSPs: Heat shock proteins
JA: Jasmonic acid
KEGG: Kyoto Encyclopedia of Genes and Genomes
qRT-PCR: Quantitative real-time PCR
RPKM: Reads per kilobases per millionreads
XT: XIANTIAN 5
ZF: ZHEFENGTIAN 2
