# Supplementary material for: Comparative transcriptome analysis reveals the transcriptional alterations in heat-resistant and heat-sensitive sweet maize (Zea mays L.) varieties under heat stress
Source: BMC Plant Biol. 2017 Jan 25;17:26. doi: 10.1186/s12870-017-0973-y (PMC5267381; doi:10.1186/s12870-017-0973-y)
Supplement: Additional file 2: — Eleven pairs of primers were designed for gene-specific transcript amplification. (DOC 28 kb) [file 12870_2017_973_MOESM2_ESM.doc]

The following primers were designed for gene-specific transcript amplification:

XM_008655452-F: 5’-TGCTCAGGAAAGACAAGGCG-3’,

XM_008655452-R: 5’-GGCCAGCACCGATAGGAAGT-3’;

XM_008675504-F: 5’-CACCCTCTTGCTCACGCGTA-3’,

XM_008675504-R: 5’-TTTCCCCCAGGTTGCACACT-3’;

XM_008680505-F: 5’-TCGTGGAAGTACGTGCTCTT-3’,

XM_008680505-R: 5’-TTAGCGACGCGTACGTAGTC-3’;

XM_008677226-F: 5’-TCCATGTTCGGCTTCTTCGT-3’,

XM_008677226-R: 5’-GTAAGCCCAGGCGTTGTTGT-3’;

NM_001154967-F: 5’-CTTCGTGCAGGCTATCGTCA-3’,

NM_001154967-R: 5’-ACGAAGTTGGTGGCGTAAGC-3’;

NM_001139328-F: 5’- TTTAGCCCGGTACCCCGAAT-3’,

NM_001139328-R: 5’-TTCTTTGTCAGCAGCGGCAC-3’;

NM_001136625-F: 5’- CCCTCGACAAGAACCTTGCA -3’,

NM_001136625-R: 5’- TTTCCCCTCGTTTTCAGCCT -3’;

NM_001137336-F: 5’- AGCCAAGGAGCTGCTCAATG -3’,

NM_001137336-R: 5’- CGGAGGAAGCGGAGGTACTT -3’;

NM_001175010-F: 5’- TTCATCGACACCTCGTCCAA -3’,

NM_001175010-R: 5’- CGCTTGACACCATCAGCAAC -3’;

XM_008671301-F: 5’- TCTCCTGCCACTTTTGCGAG -3’,

XM_008671301-R: 5’- CAATTCCGGCCTTCTTGGTT -3’;

actin-F: 5’- TGAAACCTTCGAATGCCCAG -3’,

actin-R 5’- GATTGGAACCGTGTGGCTCA -3’.
